# Analytical Study of SH Wave Scattering by a Circular Pipeline in an Inhomogeneous Concrete with Density Variation

**DOI:** 10.3390/ma16103693

**Published:** 2023-05-12

**Authors:** Zailin Yang, Chenxi Sun, Guanxixi Jiang, Yunqiu Song, Xinzhu Li, Yong Yang

**Affiliations:** 1 College of Aerospace and Civil Engineering, Harbin Engineering University, Harbin 150001, China; 2 School of Physical and Mathematical Sciences, Nanjing Tech University, Nanjing 211800, China

**Keywords:** inhomogeneous concrete, SH wave, circular pipeline, complex function method, dynamic stress concentration factor

## Abstract

In this paper, the shear horizontal (SH) wave scattering by a circular pipeline in an inhomogeneous concrete with density variation is studied. A model of inhomogeneous concrete with density variation in the form of a polynomial-exponential coupling function is established. By using the complex function method and conformal transformation, the incident and scattering wave field of SH wave in concrete are obtained, and the analytic expression of dynamic stress concentration factor (DSCF) around the circular pipeline is given. The results show that the inhomogeneous density parameters, the wave number of the incident wave and the angle of the incident wave in concrete are important factors affecting the distribution of dynamic stress around the circular pipe in concrete with inhomogeneous density. The research results can provide a theoretical reference and a basis for analyzing the influence of circular pipeline on elastic wave propagation in an inhomogeneous concrete with density variation.

## 1. Introduction

In recent years, there has been significant interest in studying the propagation of elastic waves in solids, as it is crucial for understanding wave propagation mechanisms in engineering applications such as non-destructive testing of structures and the use of new materials [1,2,3,4,5,6,7,8]. Concrete, being a common and popular engineering material, has been extensively studied for its elastic wave propagation mechanism [9,10,11,12,13,14]. In many concrete structures, circular cavity structures such as pipelines are present, and understanding their response to dynamic loads is important for engineering purposes. At present, there are many different applications for elastic wave propagation in concrete. Many studies have focused on structural damage or defects in concrete. For instance, Ziaja [15] used elastic wave propagation to monitor the state of GFRP-reinforced concrete structural members. They used PZT (lead-zirconate-titanate) sensors to record the changing state of elastic waves caused by cracks and crack propagation in GFRP reinforced concrete structures, considering the material discontinuity caused by cracks and the influence of strain field on wave propagation. Yoon [16] analyzed the applicability of the elastic wave of impact echo (IE) and evaluated six types of prestressed concrete structures using multichannel analysis of surface waves, electromagnetic waves, and shear waves. A more accurate classification method for internal defects in pipelines was proposed by using electromagnetic wave, IE, and principal component analysis (PCA). Beniwal et al. [17] proposed two different ultrasonic imaging techniques designed to use more information contained in the scattered fields for concrete using scattered elastic compression and mode conversion shear wave field modes. Guo [18] established the basic equation of elastic wave propagation in damaged concrete media based on the basic principles of classical elastic dynamics and the damage mechanics model, and derived the fundamental solution of the system. Due to the existence of damage in the structure, the wave response of concrete, including the shape, amplitude, and propagation time of ultrasonic waves in the structure, will change obviously. Additionally, many scholars have used the impact-echo method to detect defects in concrete structures [19,20]. Ali [21] described the theoretical basis of a crack detection and location method for concrete samples based on the time for elastic waves generated by crack formation to reach a set of sensors located at the sample boundary, and presented a location method based on acoustic emission detection, and developed a discretization scheme for two-dimensional elastic equations. Uenishi [22] used a two-dimensional in-plane time-harmonic elastodynamics model to analyze the effects of P wave and SV wave incidence on a circular tunnel with lining located at a finite depth from a nearly flat free surface of a homogeneous isotropic linear elastic medium. They also discussed the influence of wavelength and incidence angle, covering layer thickness, and relative compliance on the relative compliance of the linear elastic lining. The results of spalling of lining concrete, buckling of side wall reinforcement, and disengagement of subgrade from invert were given.

In practical engineering structures, the uneven density of concrete can significantly impact the mechanical properties of materials and structures. For example, Lu and Liu [23] analyzed the maximum first principal stress and mid-span deflection increment of density gradient concrete continuous rigid frame bridges under shrinkage and creep effect. Their research aimed to address the problems of excessive mid-span deflection and box girder cracking. The results showed that the effect of shrinkage and creep was reduced by a continuous rigid frame bridge with density gradient concrete. This research provided a theoretical basis for the successful application of continuous rigid-frame bridge with density gradient concrete. Not only do inhomogeneous concrete structures appear in practical projects, but the 3D printing technology of concrete is also becoming increasingly mature, making it possible to prepare concrete materials with gradually functional gradients [24]. Foamed concrete with functional gradients also plays a role in protecting the structure. For example, Strieder [25] used a simplified model to study the influence of gradient concrete material distribution on crack reduction in mass concrete structures. It also demonstrated that graded concrete may help reduce the confinement stress and weaken the risk of cracks during the hardening of concrete. Wang [26] proposed a layered graded foamed concrete-filled tensile honeycomb structure, which achieved multi-level structural protection by adjusting its overall compression deformation mode to layer-by-layer deformation mode. For example, the coagulative density of the new material UR50 ultra-early-strength concrete could reach 2600 kg/m³. This concrete is characterized by ultra-high strength, ultra-high toughness, ultra-impact resistance, and ultra-high durability. Wei et al. [27,28] also researched the mechanical properties and penetration resistance of this high-density concrete. The density of foamed concrete was relatively low, and the density span was large, ranging from 300 kg/m³ to 1600 kg/m³. Therefore, some scholars investigated the performance and structure of foamed concrete with different densities [29]. Foamed concrete backfill has been proven to be an ideal material for improving the bearing capacity of underground engineering structures, except for buried pipelines. Wang [30] studied the effect of foamed concrete backfill on improving the anti-knock performance of buried pipelines. In addition, the study of elastic wave propagation in heterogeneous concrete is of great significance. The propagation characteristics of elastic waves can be used to analyze the structural characteristics of heterogeneous concrete. Metais [31] investigated the impact of multiple scattering on the dispersion curve of phase dry surface waves by considering elastic circular inclusions in an elastic matrix. The dispersion curves were calculated using the global neighborhood algorithm and were inverted to obtain a solution for layered media with linear uniform and isotropic elastic layers. The study quantified the effect of multiple scattering on the results. As the phase velocity of surface waves does not change with frequency, a solution consisting of uniform layers was obtained through inversion. Many scholars have studied the dynamic stress response of homogeneous concrete with defects by numerical simulation or experiment. The dynamic stress response of inhomogeneous concrete with defects is rarely reported. For example, variations in density and shear modulus in inhomogeneous concrete, and structural forms of defects in concrete. These factors will affect the dynamic stress response of concrete to elastic waves. Even less research has been done on the analytical solutions to such problems. This paper aims to investigate the scattering of SH waves in inhomogeneous concrete containing a circular pipeline using complex function theory and conformal transformation proposed in Refs. [32,33,34,35,36]. In Section 2, the model of density inhomogeneous concrete and the wave field model are introduced. The governing equation is given in Section 3. In Section 4, the stress and displacement fields of the concrete are derived. In Section 5, unknown factors are solved according to boundary conditions, and the expression of dynamic stress concentration factor (DSCF) around the circular pipeline is obtained. In Section 6, the influence of reference wave number and two kinds of density inhomogeneous parameters on DSCF around the circular pipeline is discussed. Finally, Section 7 summarizes the work of this paper.

## 2. Concrete and Waves Field Model

### 2.1. Concrete Model

**Figure 1:**
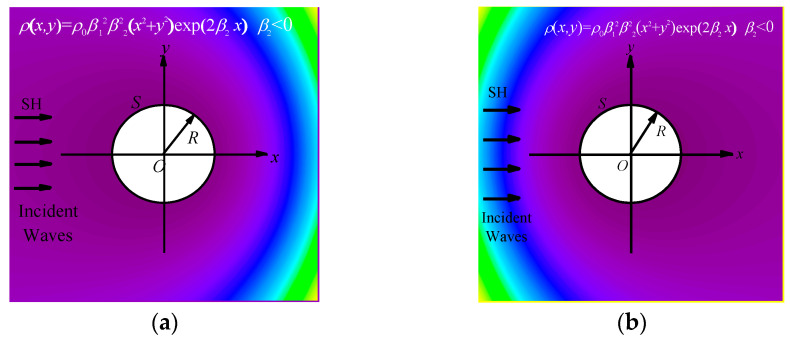
Model of inhomogeneous concrete containing a circular pipeline.

The density of concrete will change in the actual engineering structure [23]. In addition, long and thin pipelines such as drainage pipelines will also exist in concrete structures. In view of the above possible situations, this paper assumes that the density of concrete is inhomogeneous. We propose a concrete structure model in which a large volume of concrete contains a relatively small circular pipeline. It is assumed that concrete media is infinite in a two-dimensional plane, and the shear modulus of the concrete *μ* is considered to be a constant value of *μ*_0_. The density of the concrete is expressed as a polynomial and exponential coupling function, which changes with *x* and *y* in two directions simultaneously and continuously. This problem model is shown in Figure 1. The expressions of concrete density are given by the following equations.

(1) $$\rho \left(x,y\right)=\rho_{0}\cdot A\left(x,y\right)\cdot B\left(x\right),$$

(2) $$A\left(x,y\right)=\beta_{1}^{2}\beta_{2}{}^{2}(x^{2}+y^{2}),$$

(3) $$B\left(x\right)=\exp\left(2\beta_{2}x\right),$$

where $\rho_{0}$ is the reference density of the concrete, $\beta_{1}$ and $\beta_{2}$ are the inhomogeneous parameters of density; The expression of function *A* is the polynomial structure of *x* and *y*, the expression of function *B* is the exponential form.

Since the numerical value of the density of the concrete should exist and be real, neither the inhomogeneous parameters $\beta_{1}$ or $\beta_{2}$ in the density distribution function expression can be equal to 0. Moreover, the values of inhomogeneous parameters $\beta_{1}$ and $\beta_{2}$ affect the variation form of concrete density. The variation of inhomogeneous parameters $\beta_{1}$ affects the density value in the infinite concrete, while the variation of inhomogeneous parameters $\beta_{2}$ not only affects the density value, but also directly affects the density distribution in the concrete.

Then, the expression of wave number *k* is given by the following formula:

(4) $$k\left(x,y\right)=k_{0}\sqrt{A\left(x,y\right)\cdot B\left(x\right)},$$

(5) $$k_{0}=\frac{\omega_{0}}{\sqrt{\frac{\mu_{0}}{\rho_{0}}}},$$

where $k_{0}$ is the reference wave number, $\omega_{0}$ is the circular frequency.

### 2.2. SH Waves Field Model

The scattering field model is shown in Figure 1. The radius of the circular pipeline is *R*, and the center of the circular pipeline coincides with the coordinate origin *O*. The density inhomogeneous parameter $\beta_{2}$ in Figure 1a is a positive number, whereas in Figure 1b, the density inhomogeneous parameter $\beta_{2}$ is negative. The change from yellow to purple represents that the concrete density changes from large to small. It can be seen from Figure 1 that the density changes in the 2D direction, and is symmetrically distributed along the *x*-axis. Based on the symmetry of density in the concrete, the incident SH wave is assumed to incident horizontally along the *x*-axis. When $\beta_{2}$ is positive, SH waves are incident from the low density to the high density of the concrete; when $\beta_{2}$ is negative, SH waves are incident from the high density to the low density of the concrete. The incident direction of the two cases is completely opposite.

## 3. Governing Equation

In the Cartesian coordinate system, the wave equation in the concrete with inhomogeneous density is given by the following equation:

(6) $$\frac{\partial^{2}\varphi (x,y)}{\partial x^{2}}+\frac{\partial^{2}\varphi (x,y)}{\partial y^{2}}+k^{2}(x,y)\cdot \varphi (x,y)=0,$$

where φ(x,y) is the displacement in the wave field, which is the function of *x* and *y*.

Based on the complex function theory, a set of complex variables, z=x+iy and $\bar{z}=x-iy$, where introduced to transform the wave Equation (6) into the following equation:

(7) $$\frac{\partial^{2}\varphi }{\partial z\partial \bar{z}}+\frac{1}{4}k^{2}(z,\bar{z})\cdot \varphi =0,$$

where $k(z,\bar{z})$ is expressed as $k(z,\bar{z})=k_{0}\beta_{1}\beta_{2}\left|z\right|\exp\left[0.5\beta_{2}\left(z+\bar{z}\right)\right]$ in the coordinates of the complex variables.

To solve wave Equation (5), we need to introduce a new set of variables ζ and $\bar{\zeta }$

(8) $$\zeta =w(z)=\beta_{1}(z-\frac{1}{\beta_{2}})\exp(\beta_{2}z)\;,\bar{\zeta }=w(\bar{z})=\beta_{1}(\bar{z}-\frac{1}{\beta_{2}})\exp(\beta_{2}\bar{z}).$$

By introducing a new set of variables, the Helmholtz equation with variable coefficients can be transformed into the standard one, allowing for the easy derivation of analytic solutions for displacement and stress fields using the separation of variables method. The standard form of the Helmholtz equation is expressed as:

(9) $$\frac{\partial^{2}\varphi }{\partial \zeta \partial \bar{\zeta }}+\frac{1}{4}k_{0}^{2}\varphi =0.$$

## 4. Fields of Displacements and Stresses

The propagation direction of the incident wave is horizontal and the incident angle is 0°. In the ζ-plane, the displacement of the wave field can be obtained by using the Helmholtz equation in the standard form, and the expression of the incident waves $\varphi^{\left(i\right)}$ is as follows.

(10) $$\varphi^{\left(i\right)}\left(\zeta ,\bar{\zeta }\right)=\varphi_{0}\exp\left[\frac{ik_{0}}{2}\left(\zeta +\bar{\zeta }\right)\right],$$

where $\varphi_{0}$ is the amplitude of the incident wave.

In addition, a circular pipeline exists in an infinite inhomogeneous concrete, and the scattering waves $\varphi^{\left(s\right)}$ from the circular pipeline is,

(11) $$\varphi^{\left(s\right)}\left(\zeta ,\bar{\zeta }\right)=\sum_{n=-\infty }^{\infty }C_{n}H_{n}^{\left(1\right)}\left(k_{0}\left|\zeta \right|\right){\left(\frac{\zeta }{\left|\zeta \right|}\right)}^{n},$$

where $C_{n}$ are undetermined coefficients and $H_{n}^{\left(1\right)}$ is the first kind Hankel function of *n*th order.

In the concrete, the displacement field should be the superposition of incident waves and scattering waves displacement, so the displacement fields in the concrete with inhomogeneous density can be expressed as:

(12) $$\varphi_{t}=\varphi^{\left(i\right)}+\varphi^{\left(s\right)},$$

where $\varphi_{t}$ represents the total displacement field.

In the complex plane, the expression of hoop stresses and radial stresses in the concrete with inhomogeneous density is given by the following equation,

(13) $$\tau_{rz}=\mu_{0}\left(\frac{\partial \varphi }{\partial z}e^{i\theta }+\frac{\partial \varphi }{\partial \bar{z}}e^{-i\theta }\right),$$

(14) $$\tau_{\theta z}=i\mu_{0}\left(\frac{\partial \varphi }{\partial z}e^{i\theta }-\frac{\partial \varphi }{\partial \bar{z}}e^{-i\theta }\right),$$

where *r*, θ, and *z* are the cylindrical coordinates.

By introducing variables ζ and $\bar{\zeta }$, Equations (13) and (14) can be transformed into the following,

(15) $$\tau_{rz}=\mu_{0}\left(\frac{\partial \varphi }{\partial \zeta }\frac{d\zeta }{dz}e^{i\theta }+\frac{\partial \varphi }{\partial \bar{\zeta }}\frac{d\bar{\zeta }}{d\bar{z}}e^{-i\theta }\right),$$

(16) $$\tau_{\theta z}=i\mu_{0}\left(\frac{\partial \varphi }{\partial \zeta }\frac{d\zeta }{dz}e^{i\theta }-\frac{\partial \varphi }{\partial \bar{\zeta }}\frac{d\bar{\zeta }}{d\bar{z}}e^{-i\theta }\right).$$

By substituting the displacement of incident waves and scattering waves into Equations (15) and (16), the stresses field in the infinite inhomogeneous concrete in the ζ plane can be obtained,

(17) $$\tau_{rz}^{\left(i\right)}=\frac{1}{2}i\mu_{0}k_{0}\varphi_{0}\beta_{1}\beta_{2}\left[z\exp\left(\beta_{2}z+i\theta \right)+\bar{z}\exp\left(\beta_{2}\bar{z}-i\theta \right)\right]\exp\left[\frac{ik_{0}}{2}\left(\zeta +\bar{\zeta }\right)\right],$$

(18) $$\tau_{\theta z}^{\left(i\right)}=-\frac{1}{2}\mu_{0}k_{0}\varphi_{0}\beta_{1}\beta_{2}\left[z\exp\left(\beta_{2}z+i\theta \right)-\bar{z}\exp\left(\beta_{2}\bar{z}-i\theta \right)\right]\exp\left[\frac{ik_{0}}{2}\left(\zeta +\bar{\zeta }\right)\right],$$

(19) $$\tau_{rz}^{\left(s\right)}=\frac{\mu_{0}k_{0}\beta_{1}\beta_{2}}{2}\sum_{n=-\infty }^{\infty }C_{n}\left\{H_{n-1}^{\left(1\right)}\left(k_{0}\left|\zeta \right|\right){\left(\frac{\zeta }{\left|\zeta \right|}\right)}^{n-1}z\exp\left(\beta_{2}z+i\theta \right)\right.\left.-H_{n+1}^{\left(1\right)}\left(k_{0}\left|\zeta \right|\right){\left(\frac{\zeta }{\left|\zeta \right|}\right)}^{n+1}\bar{z}\exp\left(\beta_{2}\bar{z}-i\theta \right)\right\},$$

(20) $$\tau_{\theta z}^{\left(s\right)}=\frac{i\mu_{0}k_{0}\beta_{1}\beta_{2}}{2}\sum_{n=-\infty }^{\infty }C_{n}\left\{H_{n-1}^{\left(1\right)}\left(k_{0}\left|\zeta \right|\right){\left(\frac{\zeta }{\left|\zeta \right|}\right)}^{n-1}z\exp\left(\beta_{2}z+i\theta \right)\right.\left.+H_{n+1}^{\left(1\right)}\left(k_{0}\left|\zeta \right|\right){\left(\frac{\zeta }{\left|\zeta \right|}\right)}^{n+1}\bar{z}\exp\left(\beta_{2}\bar{z}-i\theta \right)\right\}.$$

## 5. Boundary Conditions and Dynamic Stress Concentration Factor of Circular Pipeline

According to the relationship between the infinite concrete and the circular pipeline, it can be determined that the stress freedom should be satisfied on the boundary of the circular pipeline. Therefore, the radial stresses should be zero on this boundary ($\left|z\right|=R$) as

(21) $$\tau_{rz}^{\left(t\right)}=\tau_{rz}^{\left(i\right)}+\tau_{rz}^{\left(s\right)}=0,\;\left|z\right|=R.$$

According to Equation (21), the radial stresses expressions of the incident and scattered waves are substituted into Equation (19), and the following expressions can be obtained,

(22) $$\sum_{n=-\infty }^{\infty }C_{n}\xi_{n}=\xi ,$$

where

(23) $$\xi_{n}=H_{n-1}^{\left(1\right)}\left(k_{0}\left|\zeta \right|\right){\left(\frac{\zeta }{\left|\zeta \right|}\right)}^{n-1}z\exp\left(\beta_{2}z+i\theta \right)-H_{n+1}^{\left(1\right)}\left(k_{0}\left|\zeta \right|\right){\left(\frac{\zeta }{\left|\zeta \right|}\right)}^{n+1}\bar{z}\exp\left(\beta_{2}\bar{z}-i\theta \right),$$

(24) $$\xi =-i\varphi_{0}\left[z\exp\left(\beta_{2}z+i\theta \right)+\bar{z}\exp\left(\beta_{2}\bar{z}-i\theta \right)\right]\exp\left[\frac{ik_{0}}{2}\left(\zeta +\bar{\zeta }\right)\right].$$

To solve the unknown coefficient $C_{n}$, multiply both sides of Equation (22) by $e^{-im\theta }$e, and perform integration over the interval of 2π to obtain Equation (25).

(25) $$\sum_{n=-\infty }^{\infty }\frac{C_{n}}{2\pi }\int_{-\pi }^{\pi }\xi_{n}e^{-im\theta }d\theta =\frac{1}{2\pi }\int_{-\pi }^{\pi }\xi e^{-im\theta }d\theta ,\left(m=0,\pm 1,\pm 2,\ldots ,\pm n\right).$$

Then the dynamic stress concentration factor around the circular pipeline in the concrete can be obtained, which is given by the following formula,

(26) $$\tau_{\theta z}^{*}=\left|\frac{\tau_{\theta z}^{\left(t\right)}}{\tau_{0}}\right|,$$

(27) $$\begin{array}{ll} \tau_{\theta z}^{\left(t\right)}= & -\left[z\exp\left(\beta_{2}z+i\theta \right)-\bar{z}\exp\left(\beta_{2}\bar{z}-i\theta \right)\right]\exp\left[\frac{ik_{0}}{2}\left(\zeta +\bar{\zeta }\right)\right]\\ & +\frac{i}{\varphi_{0}}\sum_{n=-\infty }^{\infty }C_{n}\left\{H_{n-1}^{\left(1\right)}\left(k_{0}\left|\zeta \right|\right){\left(\frac{\zeta }{\left|\zeta \right|}\right)}^{n-1}z\exp\left(\beta_{2}z+i\theta \right)+H_{n+1}^{\left(1\right)}\left(k_{0}\left|\zeta \right|\right){\left(\frac{\zeta }{\left|\zeta \right|}\right)}^{n+1}\bar{z}\exp\left(\beta_{2}\bar{z}-i\theta \right)\right\} \end{array}$$

where $\tau_{\theta z}^{\left(t\right)}=\tau_{\theta z}^{\left(i\right)}+\tau_{\theta z}^{\left(s\right)}$, $\tau_{0}=\mu_{0}k_{0}\varphi_{0}\beta_{1}\beta_{2}$.

## 6. Numerical Results and Discussion

After establishing the scattering model of elastic waves by circular pipeline in the infinite inhomogeneous concrete, the DSCFs around the circular pipeline can be obtained when SH waves propagate in the horizontal direction. The distribution and variation rule of the DSCFs around the circular pipeline are analyzed and discussed. The dimensionless variables used in the analysis are reference wave number $k_{0}R$, density inhomogeneous parameters $\beta_{1}$ and $\beta_{2}$, respectively. When other variables are the same as each other and the values of $\beta_{2}$ are opposite number to each other, the density distribution in the concrete is symmetric about the y-axis. When $\beta_{2}$ is positive, it can be considered that SH waves are incident horizontally from low density to high density in the concrete. When $\beta_{2}$ is negative, SH waves are incident horizontally from high density to low density in the concrete.

**Figure 2:**
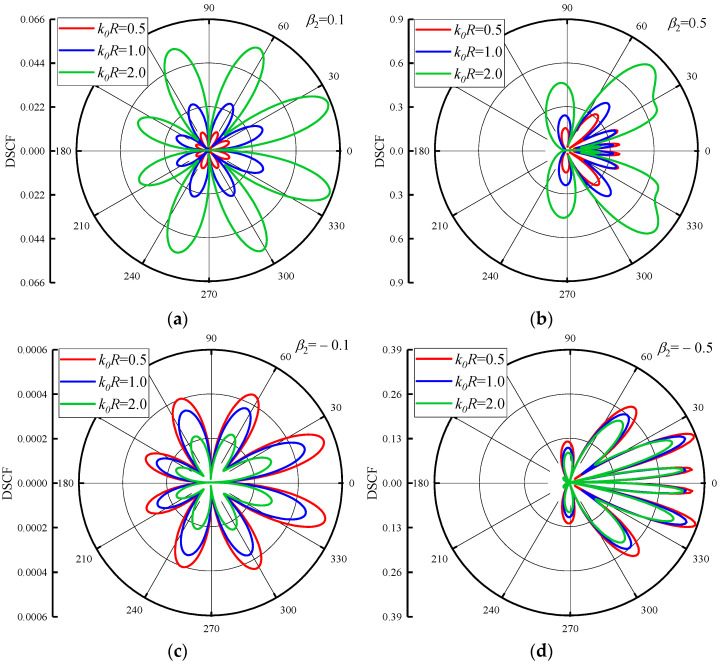
DSCF distribution around the circular pipeline with different reference wave numbers $k_{0}R$($\beta_{1}=0.5$).

To analyze the impact of different values of inhomogeneous parameters on DSCF, the values of inhomogeneous parameters adopted in this paper were based on the selected variables in the study of reference [37]. The values of variables in reference [37] can be found in Appendix A. The distribution of DSCFs around the circular pipeline is given in Figure 2 when the density inhomogeneous parameter $\beta_{1}$ = 0.5. The values of $k_{0}R$ are 0.5, 1.0 and 2.0, respectively. The density inhomogeneous parameter $\beta_{2}$ in Figure 2a,b is 0.1 and 0.5. As can be seen from the figure, since the distribution form of the inhomogeneous concrete is symmetric about the *x*-axis, and SH waves are also positively incident along the *x*-axis, the distribution of the DSCFs around the circular pipeline is also symmetric. When the reference wave numbers $k_{0}R$ become two times larger, the amplitude of the DSCF around the circular pipeline increases. It can be inferred that higher DSCF values will be obtained in the case of high-frequency waves at this density. When $\beta_{2}$ increases, the maximum value of DSCF also increases, and the extreme point of DSCF shifts toward the back wave surface, and the distribution of DSCF around the circular pipeline becomes more regular. Figure 2c,d shows that when $\beta_{2}$ is negative, the direction of density distribution in the concrete changes concerning the symmetry of the y-axis. Contrary to the results in Figure 2a,b cases, the amplitude of DSCF decreases with the increase of reference wave number $k_{0}R$, and higher DSCF values are obtained in the case of low-frequency waves. Compared with the case when $\beta_{2}$ is positive, the maximum value of DSCF when $\beta_{2}$ is negative is smaller. However, in the same way, that $\beta_{2}$ is positive, the extreme points of DSCF will shift toward the back wave surface of the circular pipeline with the increase of the absolute values of $\beta_{2}$. Thus, it can be inferred that when SH waves are incident into the concrete from two opposite directions, the distribution law and magnitude of DSCF will be changed.

By comparing the results of the DSCFs around the circular pipeline in the radial and linear terms of the density given by Jiang [37], the distribution of DSCFs around the circular pipeline given in Figure 2 shows that when the density of the concrete is distributed in the form of a polynomial-exponential coupling, more extreme points appear in the DSCF curves. The amplitudes of DSCF all appear on the surface, and there is no obvious offset with the increase of reference wave number or inhomogeneous parameters. It shows that when the density of the concrete is distributed in this form, the influence on the distribution of DSCF around the circular pipeline is more severe.

**Figure 3:**
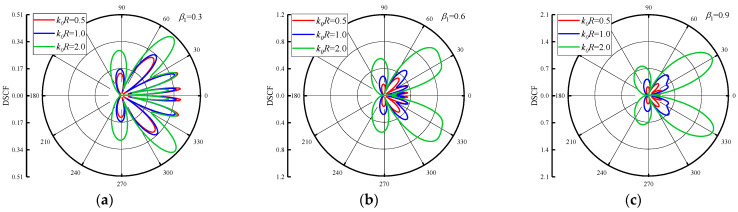
DSCF distribution around circular pipeline with different reference wave numbers $k_{0}R$ ($\beta_{2}$ = 0.5).

**Figure 4:**
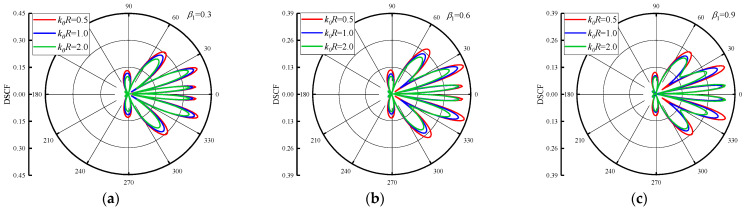
DSCF distribution around circular pipeline with different reference wave numbers $k_{0}R$ ($\beta_{2}$ = −0.5).

The values of DSCFs under different reference wave numbers $k_{0}R$ are given in Figure 3 and Figure 4. The values of $k_{0}R$ are set as 0.5, 1.0 and 2.0, respectively.

Figure 3 shows the distribution of DSCF around the circular pipeline when the $\beta_{2}$ = 0.5 and $\beta_{1}$ = 0.3, 0.6, 0.9. The DSCFs around the circular pipeline increase with larger reference wave number $k_{0}R$. When $\beta_{1}$ increases, it can be seen that the distribution of DSCF around the circular pipeline (20~40° and 320~340°) becomes regular, and the number of extreme points of DSCF around here decreases. When $k_{0}R$ and $\beta_{1}$ change, the distribution of extreme points of DSCF is dominated by the back wave surface. Figure 4 shows the DSCF distribution around the circular pipeline when the $\beta_{2}$ = −0.5. It can be seen that when $\beta_{2}$ is negative, the maximum value of DSCF decreases with the increases of $k_{0}R$, but its maximum value decreases slightly. The DSCF around the circular pipeline is complex, and there is no obvious distribution change with the increase of $\beta_{1}$, and the maximum value of DSCF is much smaller than the result in Figure 3. Thus, it can be found that when the incident direction of SH waves is from low density to high density, the distribution of DSCF in the process is more regular.

**Figure 5:**
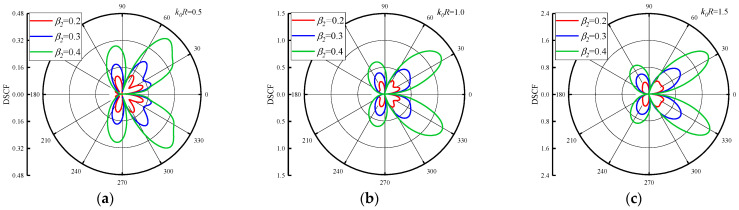
Distribution of DSCF around circular pipeline with different inhomogeneous parameter $\beta_{2}$($\beta_{2}$ = 0.2, 0.3, 0.4), ($\beta_{1}$ = 2.0).

**Figure 6:**
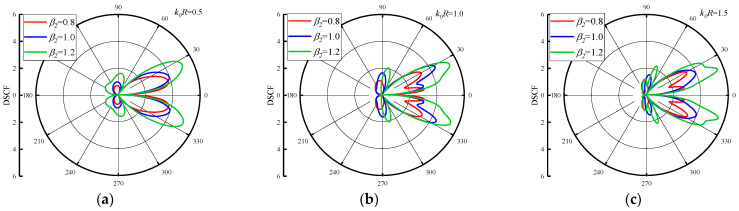
Distribution of DSCF around circular pipeline with different inhomogeneous parameter $\beta_{2}$($\beta_{2}$ =0.8, 1.0, 1.2), ($\beta_{1}$ = 2.0).

In the discussion of the influence of reference wave number $k_{0}R$ on DSCF around the circular pipeline, it is obvious that the inhomogeneous parameters $\beta_{1}$ and $\beta_{2}$ will have a significant influence on DSCF around the circular pipeline. Figure 5 shows the variation of DSCF distribution around the circular pipeline with the values of $\beta_{2}$, when the $k_{0}R$ = 0.5, 1.0 and 1.5. We set the $\beta_{1}$ = 2.0 and changed the $\beta_{2}$ = 0.2, 0.3 and 0.4. As can be seen from Figure 5, when the $\beta_{2}$ increases, the maximum value of the DSCF around the circular pipeline increases along with it. Although the values of $\beta_{2}$ are small and the increment of each change is small, the amplitude of the DSCFs around the circular pipeline is very obvious, because the change of $\beta_{2}$ has a drastic impact on the density distribution and value in the concrete. In addition, as the reference wave number $k_{0}R$ increases into an arithmetic sequence, it is found that the distribution of DSCF near 40° and 320° of the circular pipeline becomes more regular, and the number of extreme points of DSCF decreases. Figure 6 shows DSCF around the circular pipeline with the values of $\beta_{2}$ = 0.8, 1.0 and 1.2. When the value of $\beta_{2}$ is large, the maximum value of the DSCF around the circular pipeline will not change significantly with the values of $k_{0}R$ variation. Therefore, it can be inferred that when $\beta_{2}$ increases to a certain value range, the change of reference wave number has a small impact on the amplitude of DSCF around the circular pipeline. In addition with the increase of $\beta_{2}$, the distribution of DSCF near 20° and 340° of the circular pipeline gradually becomes complex, and the number of extreme points of DSCF tends to increase. These changes are completely contrary to the changes when $\beta_{2}$ is small.

**Figure 7:**
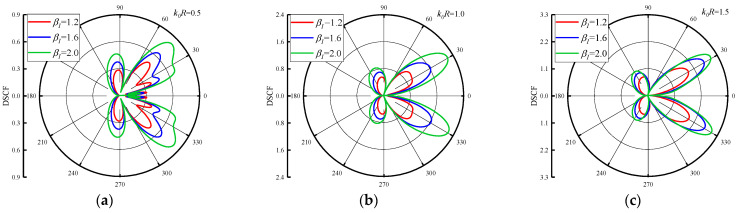
Distribution of DSCF around circular pipeline with different inhomogeneous parameters $\beta_{1}$ ($\beta_{2}$ = 0.5).

**Figure 8:**
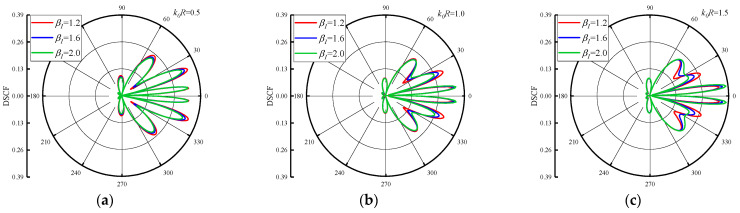
Distribution of DSCF around circular pipeline with different inhomogeneous parameters $\beta_{1}$ ($\beta_{2}$ = −0.5).

The distribution of DSCFs around the circular pipeline with $\beta_{1}$ is given in Figure 7 and Figure 8. The reference wave number $k_{0}R$ = 0.5, 1.0 and 1.5. Figure 7 set the $\beta_{2}=0.5$, and change the $\beta_{1}$ = 1.2, 1.6 and 2.0. As can be seen from Figure 7, when the $\beta_{1}$ increases, the maximum value of DSCF around the circular pipeline increases along with it. When $k_{0}R$ increases, it can be found that the distribution of DSCF near 30° and 330° of the circular pipeline changes gradually, and the number of extreme points of DSCF decreases. In addition, $k_{0}R$ increases in an arithmetic sequence, and the maximum value of DSCF increases in an arithmetic sequence. Figure 8 shows the distribution of DSCF around the circular pipeline at $\beta_{2}$ = −0.5. SH waves are incident horizontally from high density to low density in the concrete. It can be found that the distribution of DSCF around the circular pipeline is more complex than in Figure 7, in which multiple extreme points appear on the back wave surface, and when $\beta_{1}$ increases, it has little influence on the amplitude of DSCF. When $k_{0}R$ increases, the peak values of DSCF almost do not change, remaining within 0.4, which is smaller than the maximum value of DSCF in Figure 7. Only the distribution of DSCF near 30° and 330° of the circular pipeline changed slightly. It can be inferred that when SH waves are incident from high density to low density, the changes of $k_{0}R$ and $\beta_{1}$ have a very limited impact on the amplitude and distribution of DSCF around the circular pipeline, and no obvious changes will occur.

The distribution of DSCF around a circular pipeline is shown in Figure 2, Figure 3, Figure 4, Figure 5, Figure 6, Figure 7 and Figure 8, and in most cases, there are significant differences in the distribution of DSCF between 20~40° and 320~340°. In order to analyze and summarize the distribution characteristics of DSCF around the circular pipeline more intuitively, this paper selects three observation points on the circular pipe at θ = 20°, 30°, and 40°, and provides the changes in DSCF values under different variable influences in Table 1, Table 2 and Table 3.

The results of the values of DSCF at positions θ = 20°, 30°, and 40° on a circular pipeline with $\beta_{1}$ = 2.0 and $k_{0}R$ = 0.5, 1.0, 1.5 are shown in Table 1, Table 2 and Table 3. It can be visually observed that the values of DSCF at the position of θ = 30° on the circular pipeline are relatively more sensitive to changes in $\beta_{2}$ and $k_{0}R$ compared to the other two observation points, based on both horizontal and vertical comparisons of the data in the three tables. It can be inferred that changes in the parameters of inhomogeneous density and reference wavenumber have the most significant impact on the values of DSCF at the position of θ = 30° on the circular pipeline. Therefore, the position θ = 30° is selected as the observation point. The continuous variation of DSCF with $\beta_{1}$, $\beta_{2}$ and $k_{0}R$ at the observation point is analyzed and discussed.

**Figure 9:**
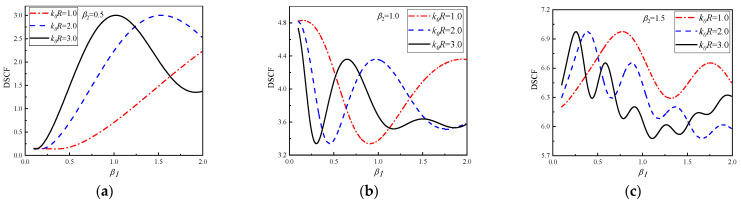

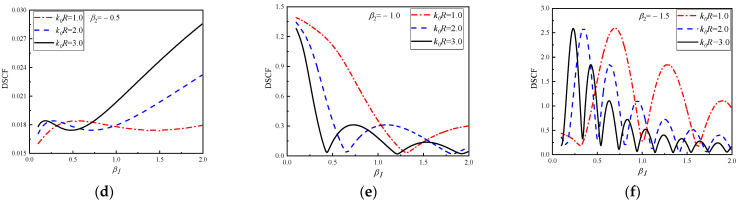
The change of DSCF at the 30° position of the circular pipeline with the inhomogeneous parameter $\beta_{1}$.

In Figure 9, DSCF changes continuously with $\beta_{1}$ at 30° of the circular pipeline with $k_{0}R$ = 1.0, 2.0 and 3.0. The $\beta_{2}$ of Figure 9a–c are 0.5, 1.0 and 1.5, respectively. It can be seen that with the continuous increase of $\beta_{1}$, the DSCF curves fluctuate significantly at the 30° of the circular pipeline. As the reference $k_{0}R$ increases, the oscillation frequency of DSCF curves increases. Another interesting point can be found in Figure 9b,c. Although the vibration frequencies of the DSCF curves in the subgraph are different, the maximum and minimum values in the same vibration cycle are approximately the same. It can be inferred that changing the density of the concrete with $\beta_{1}$ can compensate for the influence of the reference wavenumber on the amplitude of DSCF. In addition, it can be observed that when the values of $\beta_{2}$ increases, the oscillation frequency of DSCF curves will increase, but the oscillation amplitude of DSCF curves will decrease. It can be inferred that with the increase of concrete density and change of distribution, the amplitude of DSCF tends to be stable, and the fluctuation gradually decreases. The DSCF continuously changes curves with $\beta_{1}$ is given in Figure 9d–f, $\beta_{2}$ = −0.5, −1.0 and −1.5. SH waves incident horizontally from high density to low density of the concrete. It can be seen that compared with Figure 9a–c, the oscillation frequency of DSCF curves is relatively fast when the values of $\beta_{2}$ are negative, and the minimum value of each vibration period is approaching 0. Similarly, the maximum and minimum values of the three DSCF curves under different reference wave numbers are approximately the same in the same vibration period.

**Figure 10:**
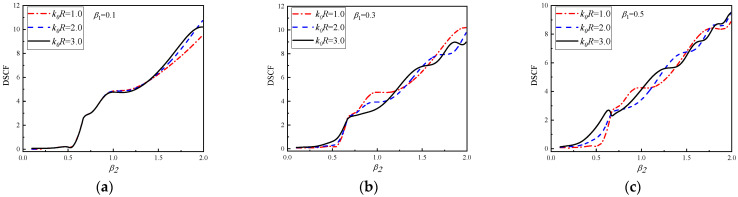
The change of DSCF at the 30° position of the circular pipeline with the inhomogeneous parameter $\beta_{2}$ (0.1 ≤ $\beta_{2}$ ≤ 2.0).

**Figure 11:**
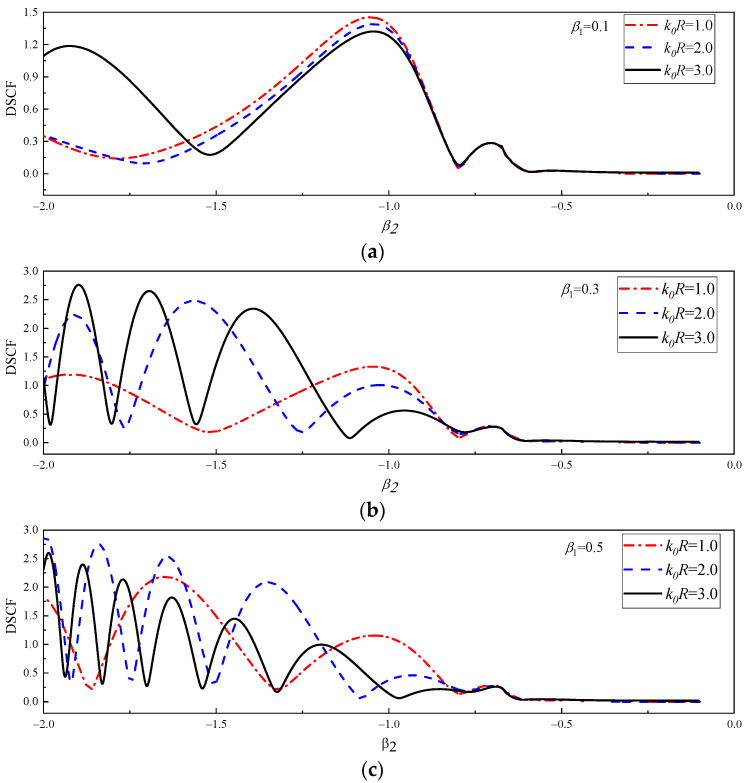
The change of DSCF at the 30° position of the circular pipeline with the inhomogeneous parameter $\beta_{2}$ (−2.0 ≤ $\beta_{2}$ ≤ −0.1).

In Figure 10 and Figure 11, the DSCF curves continuously change with $\beta_{2}$ at 30° of the circular pipeline $k_{0}R$ = 1.0, 2.0 and 3.0 are given. The values of $\beta_{1}$ are 0.1, 0.3, 0.5. The values of $\beta_{2}$ in Figure 10 are the positive of number of continuous changes with a range of 0.1~2.0, while $\beta_{2}$ in Figure 11 are the negative number of a continuous range of −2.0~−0.1.

It can be observed in Figure 10 that the overall variation trend of DSCF curves increases with the increase of $\beta_{2}$. When $\beta_{1}$ = 0.1, it can be found that within the range of 0.1 < $\beta_{2}$ < 1.5, the DSCF curves change with different values of $k_{0}R$ basically coincide without significant difference. When $\beta_{2}$ > 0.5, the DSCF curves have obvious differences. When $\beta_{1}=0.3$, the DSCF curves have obvious differences after $\beta_{2}$ > 0.5. When $\beta_{1}$ = 0.5, the DSCF curves have obvious differences after $\beta_{2}$ > 0.2. Figure 11 shows the horizontal incident of SH waves from high density to low density of concrete. It can be found that as the absolute value of $\beta_{2}$ increases, the DSCF curves at 30° of the circular pipeline have an obvious oscillation phenomenon. When the values of $k_{0}R$ and $\beta_{1}$ increase, the frequency of oscillation of DSCF curves will be higher, which is obviously different from the corresponding situation in Figure 10. When the values of $\beta_{1}$ and $\beta_{2}$ are both small, different $k_{0}R$ has little influence on the DSCF results at 30° of the circular pipeline. As the values of $\beta_{1}$ increases the absolute value of $\beta_{2}$, which causes differences between DSCF curves under different $k_{0}R$ conditions, will decrease, which is the same as the situation in Figure 10. This indicates that the density of the concrete with this density form will increase with the increases of $\beta_{1}$, so that the DSCF curves with different wave numbers appear in advance.

**Figure 12:**
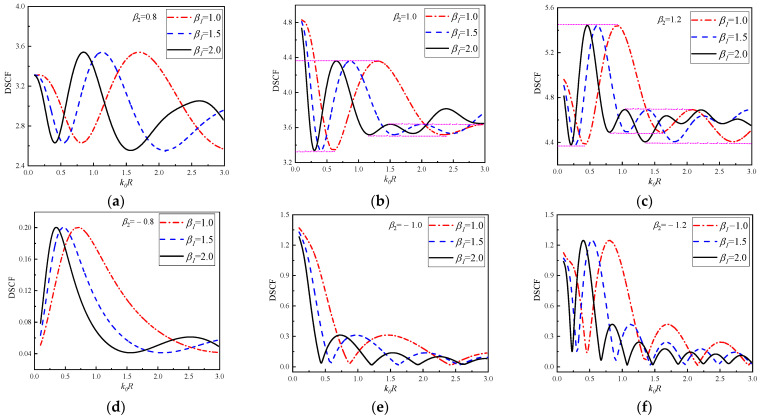
The change of DSCF at the 30° position of the circular pipeline with the reference wave number $k_{0}R$ ($\beta_{2}$ = ±0.8, ±1.0, ±1.2).

In Figure 12, the continuous change of DSCF curves with $k_{0}R$ at 30° of circular pipeline is given. In Figure 12a–c, $\beta_{1}$ = 1.0, 1.5 and 2.0, $\beta_{2}$ = 0.8, 1.0, 1.2. It can be observed that SH waves are incident horizontally from low density to high density of the concrete. It can be observed in Figure 12 that with the continuous change of $k_{0}R$, the vibration amplitude of DSCF curves decreases. When the values of $\beta_{1}$ and $\beta_{2}$ increase, the oscillation frequency of DSCF curves will increase. In Figure 12d–f, the values of $\beta_{2}$ = −0.8, −1.0 and −1.2, SH waves are incident horizontally from the high-density to the low-density direction of the concrete. In this case, compared with the case in Figure 12a–c, the maximum value of DSCF curves is less than, and the minimum points on the DSCF curves are all approaching 0. It can be found that similar to the continuous change of DSCF with $\beta_{1}$ the maximum and minimum values of different DSCF curves in the same vibration period remain the same regardless of the values of $\beta_{1}$ in each subgraph of Figure 12.

**Figure 13:**
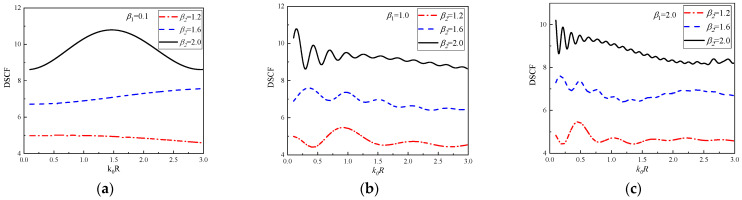
The change of DSCF at the 30° position of the circular pipeline with the reference wave number $k_{0}R$ ($\beta_{1}$ = 0.1, 1.0, 2.0).

In Figure 13, the continuous changes of DSCF curves with $k_{0}R$ at 30° of the circular pipeline are given. $\beta_{2}$ = 1.2, 1.6, and 2.0, $\beta_{1}$ = 0.1, 1.0 and 2.0. It can be observed that with the increase of $\beta_{2}$, the peak value of DSCF also increases, the oscillation of DSCF curves will appear earlier and the oscillation frequency will be higher. At the same time, the larger $\beta_{2}$ is, the closer the occurrence time of the maximum values of DSCF at the position of 30° is to the quasi-static condition ($k_{0}R$ = 0.1). With the continuous increase of $k_{0}R$, the amplitude of DSCF curves decreases. In addition, larger $\beta_{1}$ causes faster oscillation frequency of DSCF curves. Interestingly, even when the value of $\beta_{1}$ changes, the DSCF curves with $\beta_{2}$ = 1.2 always have an amplitude range of 4 to 6, the DSCF curves with $\beta_{2}$ = 1.6 always have an amplitude range of 6 to 8, and the DSCF curves with $\beta_{2}$ = 2.0 always have an amplitude range of 8 to 11.

## 7. Conclusions

In this paper, the scattering of SH waves by circular pipeline in inhomogeneous concrete with polynomial-exponential coupling density distribution is studied based on the method of complex variable function. The analytical solution of dynamic stress concentration around circular pipeline is derived under this type of concrete with density variation. This paper discusses the effect of different dimensionless parameters on the distribution of DSCF around the circular pipeline. It provides a theoretical reference and a basis for analyzing the influence of defects on wave propagation in an inhomogeneous concrete with density variation. The specific conclusions are as follows:

(1) When the density inhomogeneous parameters $\beta_{1}$, $\beta_{2}$ and the reference wave number $k_{0}R$ change, all the maximum values of DSCF always appear on the back wave surface of the circular pipeline. In most cases, the maximum value of DSCF is concentrated within the range of 20~40° and 320~340° at the position of circular pipeline, and the number of extreme points of DSCF in this range also changes significantly.
(2) When the value of $\beta_{2}$ is used to change the incident angle of SH wave, the peak values of DSCF around the circular pipeline when the values of $\beta_{2}$ is positive are much higher than that when $\beta_{2}$ is negative. Meanwhile, the distribution of DSCFs around the circular pipeline is more regular.
(3) At 30° of the circular pipeline, with the dimensionless parameter increasing, the DSCF values at this position will have an obvious oscillation phenomenon. At this position, when $\beta_{2}$ remains unchanged, $\beta_{1}$ and $k_{0}R$ changes, the maximum and minimum values of different DSCF curves in the same fluctuation cycle are the same. When $\beta_{2}$ is negative, the DSCFs at the position of 30° are higher than that of positive $\beta_{2}$.

The conformal transformation method used in this paper requires a high level of expression for the non-uniformity of concrete density, which is not yet achievable in reality. Although the inhomogeneous concrete model we presented does not exist in reality, we hope that in the future, the changing form and structural model of concrete density we proposed can be applied to concrete materials, and our research method can be applied to the elastic dynamic research of other inhomogeneous concrete forms.

## Figures and Tables

**Table 1:** The values of DSCF at different positions of circular pipeline with $k_{0}R$ = 0.5 ($\beta_{1}$ = 2.0).

$k_{0}R$	$\beta_{2}$	DSCF (θ = 20°)	DSCF (θ = 30°)	DSCF (θ = 40°)
0.5	0.2	0.1349	0.0971	0.0562
	0.3	0.1846	0.1558	0.1810
	0.4	0.2520	0.3571	0.3985
	0.5	0.6116	0.6885	0.7974

**Table 2:** The values of DSCF at different positions of circular pipeline with $k_{0}R$ = 1.0 ($\beta_{1}$ = 2.0).

$k_{0}R$	$\beta_{2}$	DSCF (DSCF (θ = 20°)	DSCF (θ = 30°)	DSCF (θ = 40°)
1.0	0.2	0.3018	0.2555	0.2124
	0.3	0.4973	0.5548	0.6070
	0.4	0.8678	1.2262	1.2294
	0.5	1.6774	2.2196	1.9365

**Table 3:** The values of DSCF at different positions of circular pipeline with $k_{0}R$ = 1.5 ($\beta_{1}$ = 2.0).

$k_{0}R$	$\beta_{2}$	DSCF (θ = 20°)	DSCF (θ = 30°)	DSCF (θ = 40°)
1.5	0.2	0.4653	0.4670	0.4509
	0.3	0.8378	1.0856	1.1452
	0.4	1.4758	2.0635	1.9871
	0.5	2.0386	2.9974	2.4527

## Data Availability

Not applicable.

## Appendix A. An Example of Choosing Values for Inhomogeneous Density Parameter and Reference Wave Number

Reference [37] presents an inhomogeneous medium with a circular cavity, and its density expression is given as:

(A1) $$\rho =\rho_{0}\left[4\alpha^{2}\left(x^{2}+y^{2}\right)+4\alpha \beta x+\beta^{2}\right],$$

where, α and β represent inhomogeneous parameters. When β/α < 5, the density of the medium is considered to vary continuously in two dimensions.

In the example, in order to analyze the variation of DSCF around a circular cavity under different reference wave numbers, reference wave numbers of 0.1, 0.5, 1.0, and 2.0 were selected. Therefore, in this article, we selected reference wave numbers of 0.5, 1.0, 1.5, and 2.0.

In the process of selecting values for inhomogeneous parameters in this example, it was controlled within the range of β/α < 5. The selected values for α and β are listed in Table A1.

**Table A1:** The values of α and β.

	α	β
Case 1	1.0	0.4
Case 2		0.5
Case 3		0.6
Case 4		1.0
Case 5		1.8
Case 6		2.0
Case 7	0.4	1.0
Case 8	0.5	
Case 9	0.6	
Case 10	1.8	
Case 11	2.0	

Although the density variation form we provide is different from the density form in the example, we still give the range of variation for $\beta_{1}$ values from 0.1 to 2.0 and $\beta_{2}$ values from −2.0 to −0.1 and 0.1 to 2.0. However, it should be noted that the selection of values for inhomogeneous parameters can affect the accuracy of the results, and different values may be more suitable for different applications.

To illustrate the impact of inhomogeneous density and reference wave number values on the results, we analyzed the DSCF variation around a circular pipeline using different values for these parameters. We found that the selection of the density function exponent ($\beta_{2}$ value) has a relatively large impact on the results compared to the values of $\beta_{1}$ and reference wave number. This is because the selection of beta value can not only change the distribution form of density in the material, but also change the direction of density distribution.

In summary, our research results indicate the importance of carefully selecting the parameters used in inhomogeneous density concrete models, and emphasize the need for further research to better understand the impact of inhomogeneous density parameters and reference wave numbers on the DSCF obtained from these models.
